# *Sophora flavescens* protects against mycobacterial Trehalose Dimycolate-induced lung granuloma by inhibiting inflammation and infiltration of macrophages

**DOI:** 10.1038/s41598-018-22286-w

**Published:** 2018-03-02

**Authors:** Dehua Liu, Ben Chung-Lap Chan, Ling Cheng, Miranda Sin-Man Tsang, Jing Zhu, Chun-Wai Wong, Delong Jiao, Helen Yau-Tsz Chan, Ping Chung Leung, Christopher Wai-Kei Lam, Chun Kwok Wong

**Affiliations:** 1 Institute of Chinese Medicine, The Chinese University of Hong Kong, Hong Kong, China; 2 State Key Laboratory of Phytochemistry and Plant Resources in West China, The Chinese University of Hong Kong, Hong Kong, China; 3 Department of Chemical Pathology, The Chinese University of Hong Kong, Hong Kong, China; 4 State Key Laboratory of Quality Research in Chinese Medicine, Macau Institute for Applied Research in Medicine and Health, Macau University of Science and Technology, Taipa, Macau China; 5 Li Dak Sum Yip Yio Chin R & D Centre for Chinese Medicine, The Chinese University of Hong Kong, Hong Kong, China

## Abstract

The immune system responds to *Mycobacterium tuberculosis* (MTB) infection by forming granulomas to quarantine the bacteria from spreading. Granuloma-mediated inflammation is a cause of lung destruction and disease transmission. *Sophora flavescens* (SF) has been demonstrated to exhibit bactericidal activities against MTB. However, its immune modulatory activities on MTB-mediated granulomatous inflammation have not been reported. In the present study, we found that flavonoids from *Sophora flavescens* (FSF) significantly suppressed the pro-inflammatory mediators released from mouse lung alveolar macrophages (MH-S) upon stimulation by trehalose dimycolate (TDM), the most abundant lipoglycan on MTB surface. Moreover, FSF reduced adhesion molecule (LFA-1) expression on MH-S cells after TDM stimulation. Furthermore, FSF treatment on TDM-activated lung epithelial (MLE-12) cells significantly downregulated macrophage chemoattractant protein (MCP-1/CCL2) expression, which in turn reduced the *in vitro* migration of MH-S to MLE-12 cells. In addition, FSF increased the clearance of mycobacterium bacteria (*Mycobacterium aurum*) in macrophages. FSF mainly affected the Mincle-Syk-Erk signaling pathway in TDM-activated MH-S cells. In TDM-induced mouse granulomas model, oral administration with FSF significantly suppressed lung granulomas formation and inflammation. These findings collectively implicated an anti-inflammatory role of FSF on MTB-mediated granulomatous inflammation, thereby providing evidence of FSF as an efficacious adjunct treatment during mycobacterial infection.

## Introduction

Mycobacteria, including *Mycobacterium tuberculosis* (MTB) causing pulmonary tuberculosis, are one of the most notorious pathogens that are difficult to be treated. Macrophages are the primary innate immune effector cells during MTB infection^1^. In response to evasive mycobacterial infection, the host can induce granulomas to quarantine and restrict bacteria from expansion^2^. In an immunocompetent host, a small granuloma-mediated inflammation can be resolved and thus the infection is controlled. However, granulomas in an immunocompromised host can result in necrotic granulomatous inflammation to cause extensive tissue damage^3^. A granuloma is mostly composed of activated macrophages and few surrounding T lymphocytes^4^. A panel of cytokines induced by interaction of MTB with macrophages can act on other cells within the granuloma and modulate its environment^5^. Therefore, proper regulation of activated macrophages is crucial for the modulation of granuloma-mediated inflammation and containment of mycobacteria infection.

Various bacterial pathogen-associated molecular patterns (PAMPs) are known to be involved in MTB pathogenesis, upon interacting with pattern recognition receptors (PRR). Among the MTB cell wall components, trehalose 6,6-dimycolate (TDM), constituting 90–95% of the outer membrane lipids, is the most abundant extractable lipid produced by virulent MTB^6^. TDM has been shown to possess immunostimulatory properties during the pathogenesis of MTB infection, including granuloma genesis^7–9^. TDM induces pulmonary granuloma formation and inflammation in mice by stimulating production of pro-inflammatory and T helper type 1 (Th1)-related cytokines such as tumor necrosis factor (TNF)-α, which represents many aspects of natural MTB infection in lungs (9–13). Taken together, the TDM mouse model provides a useful tool for investigating the response of granulomas with different treatments *in vivo*.

Increased production and release of chemokines and cytokines are crucial for the recruitment of pro-inflammatory cells and their aggregation to form granulomas^10^. MTB and its associated components have been shown to stimulate mononuclear phagocytes *in vitro* to release inflammatory interleukin (IL)-1β, TNF-α and IL-6^11^. Animal models of tuberculosis (TB) also demonstrated the presence of these cytokines in granulomas^12^. It has been demonstrated the elevated levels of inflammatory chemokines, including CCL2, CCL5, CXCL8 and CXCL10, in serum and bronchial alveolar lavage (BAL) of TB patients compared to healthy controls^13–16^. Therefore, interventions that modulate these inflammatory cytokines and chemokines may ameliorate the pathogenesis caused by MTB infections.

The roots of *Sophora flavescens* (SF) have been used in traditional Chinese medicine for the treatment of infectious diseases^17^, cancer^18^, and inflammatory disorders^19^. Moreover, its flavonoids exerted anti-inflammatory and anti-arthritic activities^20^. The *in vivo* and *in vitro* suppression of allergic reactions of these flavonoids has also been reported^21^. SF has been shown to possess anti-bacteria activities, e.g. by inhibiting the growth of MTB. However, the effects of SF on MTB-mediated granulomas, especially on granuloma-mediated chronic inflammation, have not been reported. In addition, the cellular mechanisms of SF, including its modulating effect on pro-inflammatory molecules during MTB infection, have yet to be elucidated. Among various components isolated from the roots of SF, prenylated flavonoids of *Sophora flavescens* (FSF), which contain sophoraflavanone G, kuraridin and kurarinone, were strongly suggested to exert anti-inflammatory activities^22–24^. Another main active ingredients of SF, the alkaloids, can exhibit toxic effects on mammalian animal model and some of alkaloid compounds have been used for cancer therapy^20^. Therefore, in the present study, the alkaloid-free FSF was prepared and its effect on MTB-mediated granulomatous inflammation together with its underling mechanisms was investigated.

## Results

### Standardization of herbal extracts

**Figure 1:**
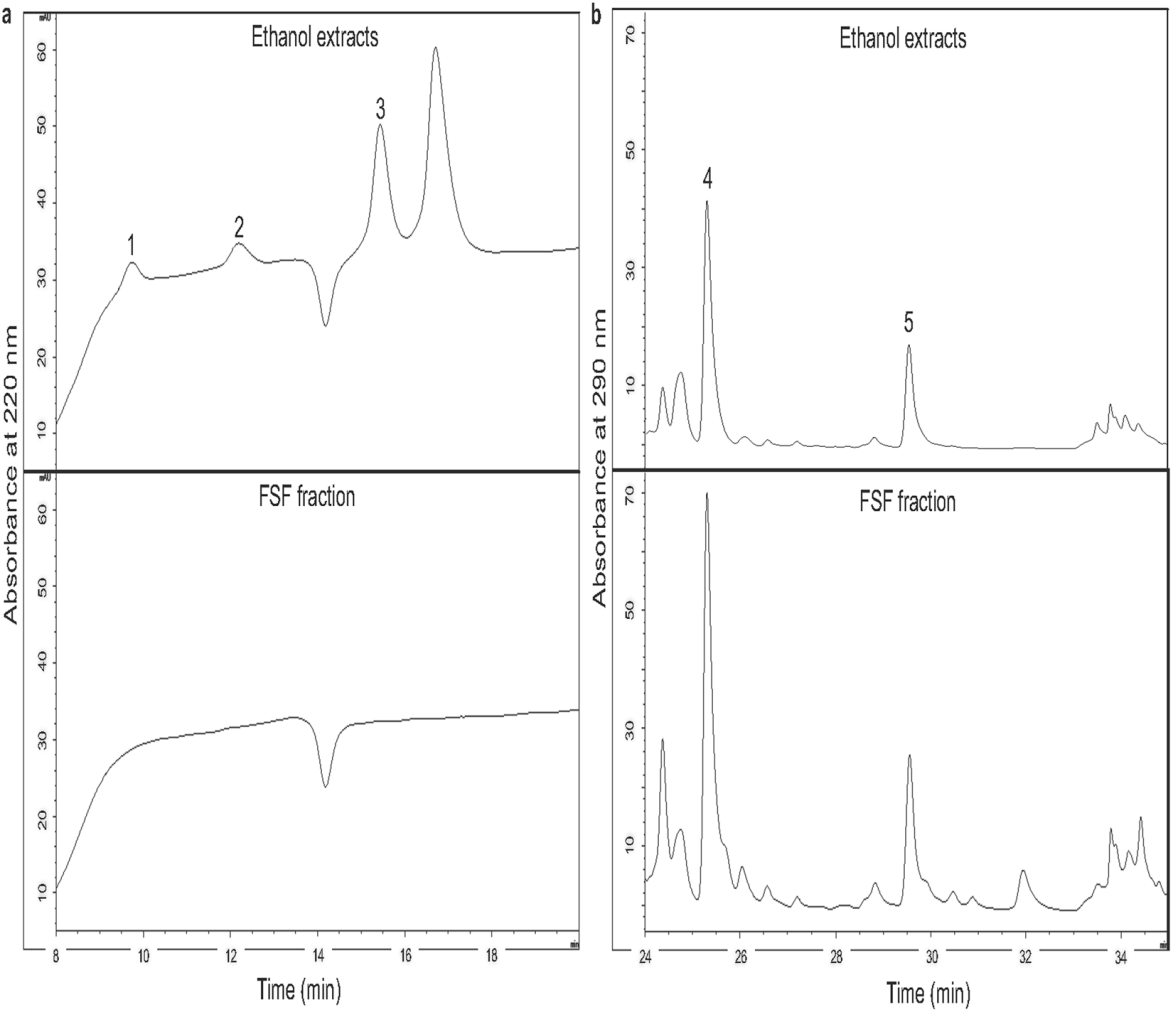
HPLC analysis of major compounds in the ethanol and alkaloid-free flavonoid (FSF) fractions. Five standard compounds, matrine (peak 1), sophoridine (peak 2), oxymatrine (peak 3), kurarinone (peak 4) and sophoraflavanone G (peak 5) were detected at 220 nm (Fig. 1a) and 290 nm (Fig. 1b), respectively.

Alkaloid-free FSF extracts were prepared according to a previously published method^20^. The extraction efficiency was 13.9 ± 2.6% and 2.4 ± 0.8% for total SF and FSF extracts, respectively (mean ± SD, n = 3). HPLC analysis was chosen to control the quality of herbal extracts by the assessment of chemical markers. Five typical chemical markers were selected: matrine, sophoridine and oxymatrine which are alkaloids, and kurarinone and sophoraflavanone G that are flavonoids. A typical HPLC chromatogram is shown in Fig. 1. No detection of alkaloids in FSF fraction was confirmed (Fig. 1a, lower panel). The concentrations of sophoraflavanone G and kurarinone in FSF were found to increase by approximately 3 times after excluding the alkaloid components, i.e. from 0.7 and 2.1% to 2.1 and 7.2%, respectively (Fig. 1b and Table s1).

### FSF inhibited pro-inflammatory cytokine/chemokine release from TDM-activated murine alveolar macrophages

**Figure 2:**
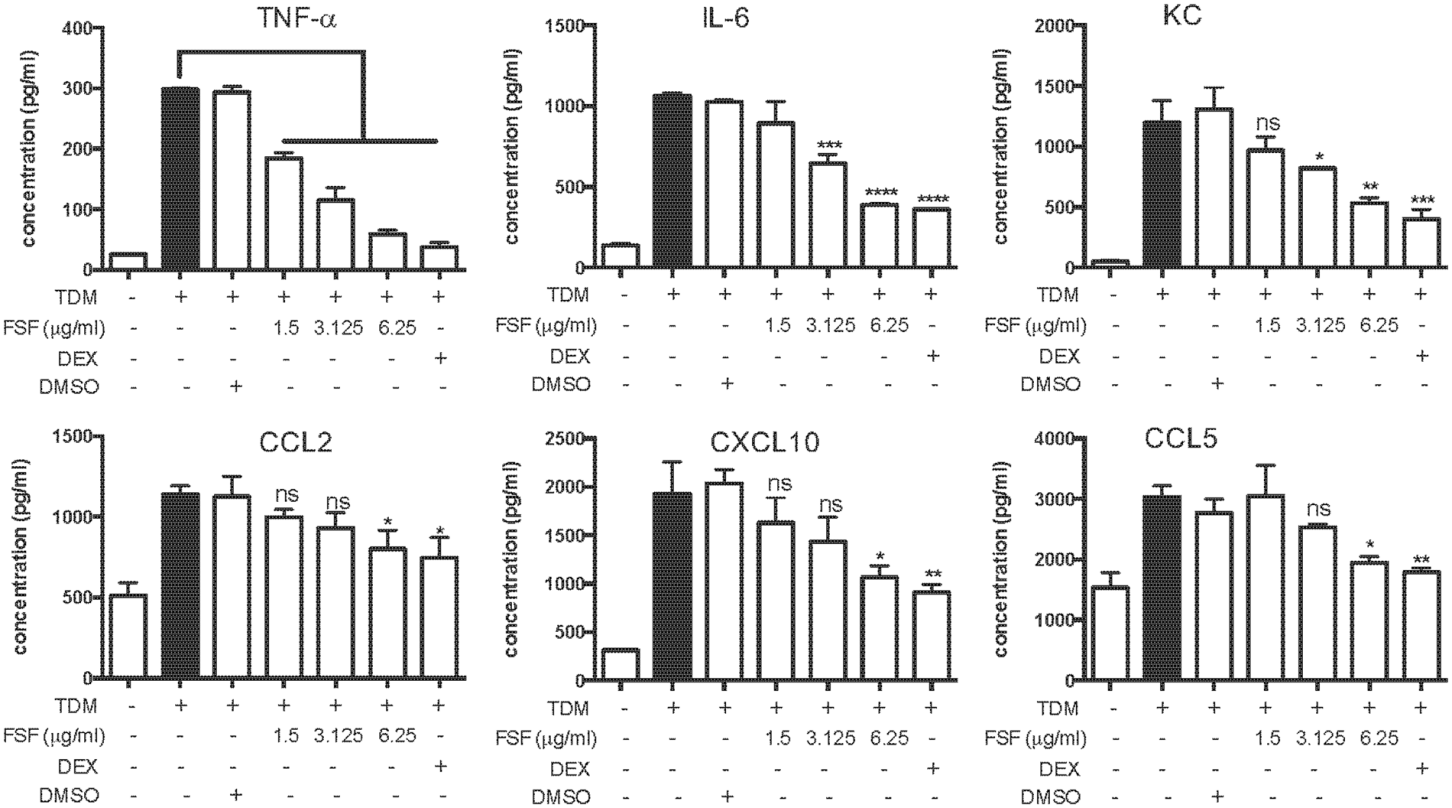
*In vitro* effects of FSF on the suppression of cytokines/chemokines production of TDM activated MH-S cells. Supernatants were collected after 20 h incubation and the release of TNF-α, IL-6, KC, CCL2, CXCL10 and CCL5 was determined. DEX, dexamethasone; ns, non-significant. Results are shown as arithmetic mean plus SD of triplicate independent experiments. ns, P > 0.05; *P < 0.05; **P < 0.01; ***P < 0.001; and ****P < 0.0001 vs. TDM stimulation group.

Since macrophages are the main innate immune cells that respond to MTB infection for the formation of granulomas in mouse lungs, we analyzed *in vitro* effects of FSF on TDM-stimulated macrophages. The cytotoxicity and optimal dose of FSF in MH-S cells were determined by the MTT assay (Fig. s1a). Accordingly, FSF (1.5, 3.125, or 6.25 μg/ml) without significant cytotoxicity was adopted for the *in vitro* experiments. As illustrated in Fig. 2, the release of cytokines (TNF-α and IL-6) and chemokines (KC, CCL2, CCL5 and CXCL10) of TDM-activated MH-S cells were significantly reduced by FSF treatment in a dose-dependent manner (Fig. 2).

### FSF inhibited pro-inflammatory cytokine/chemokine release from separately co-cultured murine alveolar macrophages/epithelial cells upon TDM stimulation

**Figure 3:**
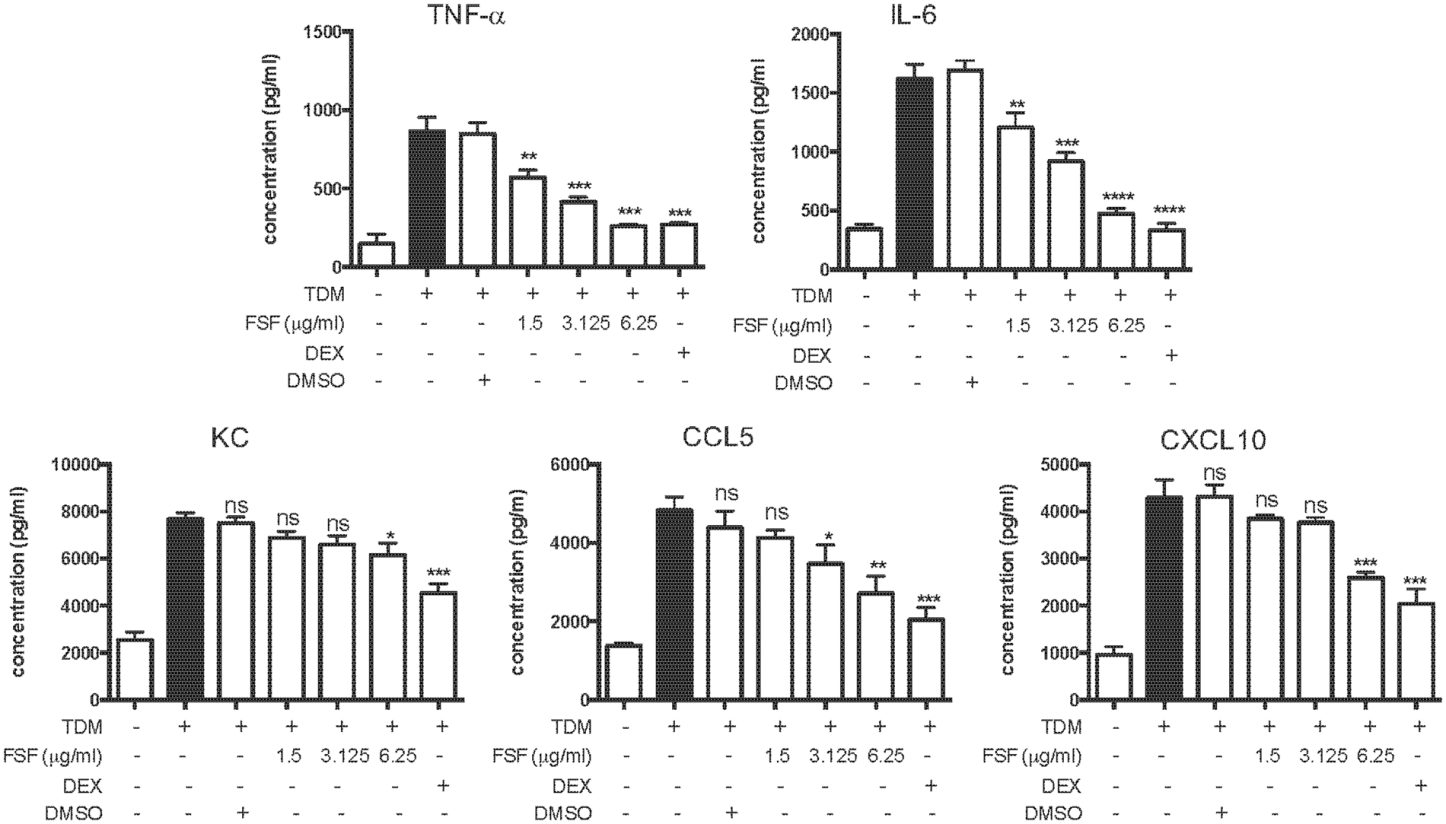
FSF inhibited release of pro-inflammatory cytokines/chemokines from co-cultured MH-S/MLE-12 cells (separated by transwells) upon stimulation of TDM. Secretion of TNF-α, IL-6, KC, CCL2, CXCL10 and CCL5 by macrophage-epithelial cell co-culture using transwells (0.4 μm pore size) following TDM stimulation with or without treatment of FSF. The supernatants in the lower and upper chamber were collected, pooled and tested after 20 h incubation. DEX, dexamethasone; ns, non-significance. Results are shown as arithmetic mean plus SD of triplicate independent experiments. ns, P > 0.05; *P < 0.05; **P < 0.01; ***P < 0.001 and ****P < 0.0001 vs. TDM stimulation group.

MH-S macrophages and MLE-12 epithelial cells, which are widely used cell culture models for studying respiratory host defense and immunity^25,26^, were chosen for investigating the anti-inflammatory activity of FSF *in vitro*. Cell viability for both cells was assessed by the MTT assay. As shown in Fig. s1a and b, cell viabilities were not decreased by FSF treatment at concentrations up to 6.25 μg/ml for both cell lines. Therefore, FSF (1.5, 3.125 or 6.25 μg/ml) was adopted for the treatment of cells in the following study. Two co-culture systems including the use of transwell (0.4 μm pore size) and transwell-free methods were employed for this assay. FSF significantly suppressed cytokines (TNF-α and IL-6) and chemokines (KC, CCL2, CCL5 and CXCL10) release in the transwell co-culture system in a dose dependent manner (Fig. 3). For co-cultured cells without transwell, no suppression of both cytokines (TNF-α and IL-6) and chemokines (KC, CCL2, CCL5 and CXCL10) were observed for FSF even at the highest non-cytotoxic concentrations (6.25 μg/mL, Fig. s2). In contrast, unlike FSF, the administration of synthetic corticosteroid dexamethasone suppressed the release of these cytokines and chemokines in both culture systems. This may indicate that soluble mediators^27^, rather than direct intercellular contact, caused the suppression of TDM-induced pro-inflammatory response by FSF in co-culture system. FSF may exert distinct anti-inflammatory mechanisms from that of dexamethasone.

### FSF suppressed *in vitro* migration of MH-S cells by inhibiting the expression of adhesion molecule and macrophage chemoattractant

**Figure 4:**
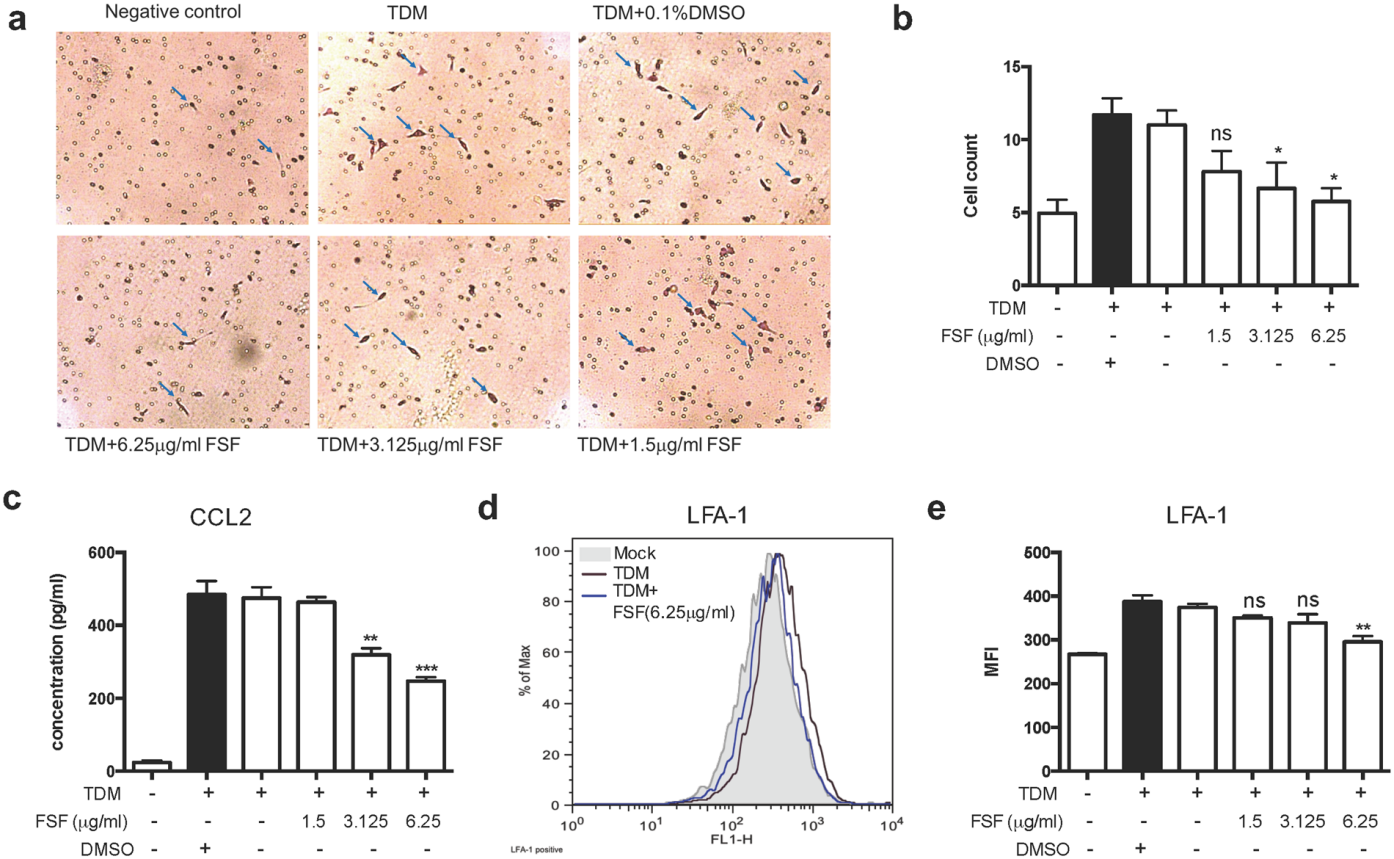
FSF suppressed the migration of MH-S cells to MLE-12 cells *in vitro*. (**a**) transwell assay for MH-S cells plated on the upper cell culture inserts, with culture medium from unstimualted-MLE-12 cells, stimulated-MLE-12 cells with or without treatment of FSF in the lower chambers. Cells on the lower surface of the transwell membrane were stained with crystal violet solution and (**b**) quantified under microscope (n = 4 per group). Arrow heads indicate migrated cells on the lower membrane of transwell inserts. (**c**) Secretion of CCL2 in the culture supernatants from TDM stimulated MLE-12 cells with or without treatment of FSF for 48 h was determined. (**d**) TDM activated MH-S cells were cultured with or without FSF treatment for 20 h. Surface staining of LFA-1 were then performed. Representative histograms of cell surface staining of LFA-1 are shown from triplicate independent experiments showing essentially similar results. Grey area represents un-stimulated cell staining; blue line represents FSF (6.25 μg/ml) treated cells with TDM stimulation; Black line represents TDM stimulation control. (**e**) Quantitative results of surface LFA-1 expression on MH-S cells are presented as MFI (mean fluorescence intensity). Results are shown as arithmetic mean plus SD of triplicate independent experiments. ns, P > 0.05; *P < 0.05; **P < 0.01 and ***P < 0.001 vs. TDM stimulation group.

To demonstrate the effect of FSF on the inhibition of macrophage migration, *in vitro* transwell (8 μm pore size) assay was performed. TDM activated MLE-12 cells were treated with FSF at various concentrations for 48 hours and the MLE-12 conditioned supernatants were collected for the macrophage migration assay. Compared with the untreated group, administration of FSF suppressed migration of MH-S cells to MLE-12 conditioned medium in a dose-dependent manner, with significant inhibition (∼50%) at 3.125 and 6.25 μg/ml (Fig. 4a and b).

Monocyte chemoattractant protein-1 (MCP-1/CCL2) is one of the key chemokines that regulate the migration and infiltration of monocytes/macrophages^28^. To investigate whether FSF suppressed the expression of CCL2 in TDM activated MLE-12 cells, the release of CCL2 in the supernatant of FSF treated MLE-12 cells was analyzed by Milliplex assay. Results showed that the expression of CCL2 was found to be significantly suppressed by FSF treatment at 3.125 and 6.25 μg/ml (P < 0.01 and P < 0.001, respectively, Fig. 4c). Thus, the inhibition of CCL2 release from MLE-12 cells might account for the decreased migration of MH-S cells to MLE-12 cells.

Leukocyte function associated antigen-1 (LFA-1; αLβ2; CD11a/CD18) activation is crucial for inflammatory cell adhesion and migration^29^. We herein investigated the effect of FSF on the cell surface expression of LFA-1 on TDM activated MH-S cells. As shown in Fig. 4d and e, the expression of LFA-1 increased significantly upon stimulation by TDM, and treatment of FSF (6.25 μg/ml) substantially suppressed LFA-1 expression (all P < 0.05). These results suggested that the inhibition on LFA-1 expression on macrophages by FSF may account for the reduced migration of MH-S to the inflamed MLE-12 cells.

### FSF suppressed the mycobacteria survival in macrophages

**Figure 5:**
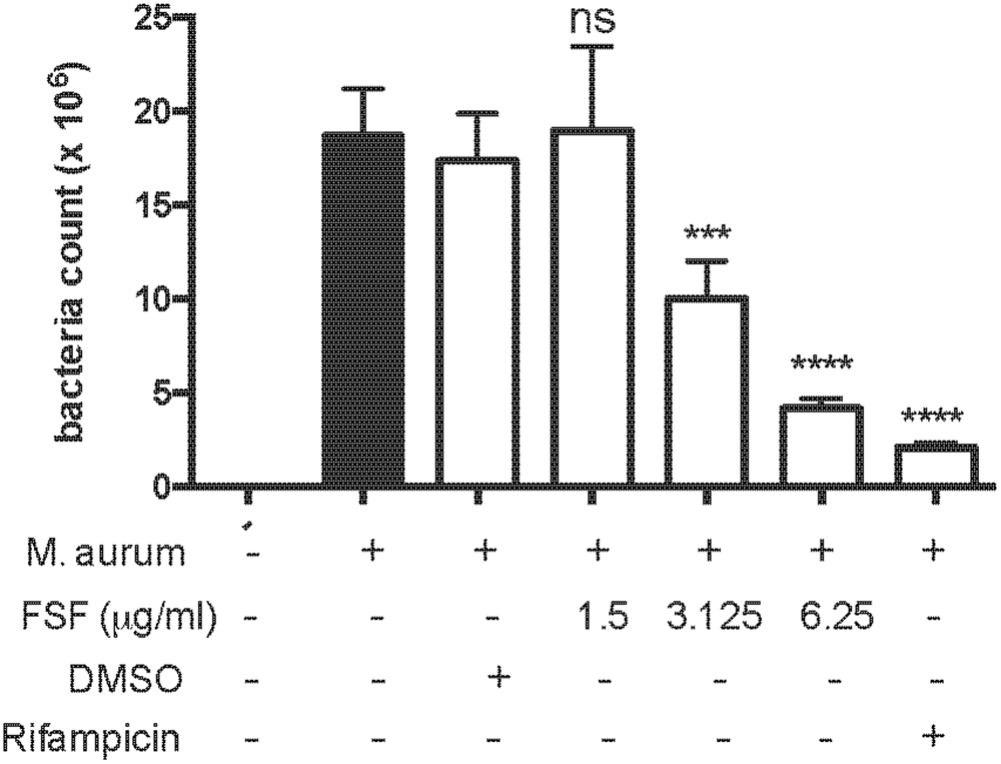
FSF increased the clearance of mycobacterial in lung alveolar macrophages. After 3 hours of M. aurum infection, MH-S cells were washed with RPMI 1640 and new complete culture medium with different concentrations of FSF were added into each well, rifampicin (1 μg/ml) served as a positive control. The cells were harvested after 24 hours’ incubation and intracellular survival of M. aurum was evaluated by cfu counting. ns, ***P < 0.001 and ****P < 0.0001 vs. M. aurum infection group.

Previous study has reported the anti-microbial activities of SF^30,31^, however, the effect of SF on the antimicrobial property of macrophages has not been evaluated. In this study, an intracellular growing *Mycobacterium aurum Tsukamura* (M. aurum) (ATCC) strain was employed. M. aurum is a fast-growing and non-pathogenic organism for investigating agents against *Mycobacterium tuberculosis*^32–34^. Herein, we administrated FSF on M. aurum infected macrophages and assessed intracellular survival of bacteria after 24 hrs. As shown in Fig. 5, FSF (3.125 and 6.25 μg/ml, P < 0.001) exerted significant inhibition of M. aurum survival in macrophages. Meanwhile, a significant inhibition of M. aurum survival by rifampicin (1 μg/ml), a widely used front-line anti-TB drug, was also observed. Taken together, this result suggested the anti-intracellular mycobacterial activity of FSF in macrophages.

### FSF affected Mincle-Syk-Erk signaling pathway in TDM activated MH-S cells

**Figure 6:**
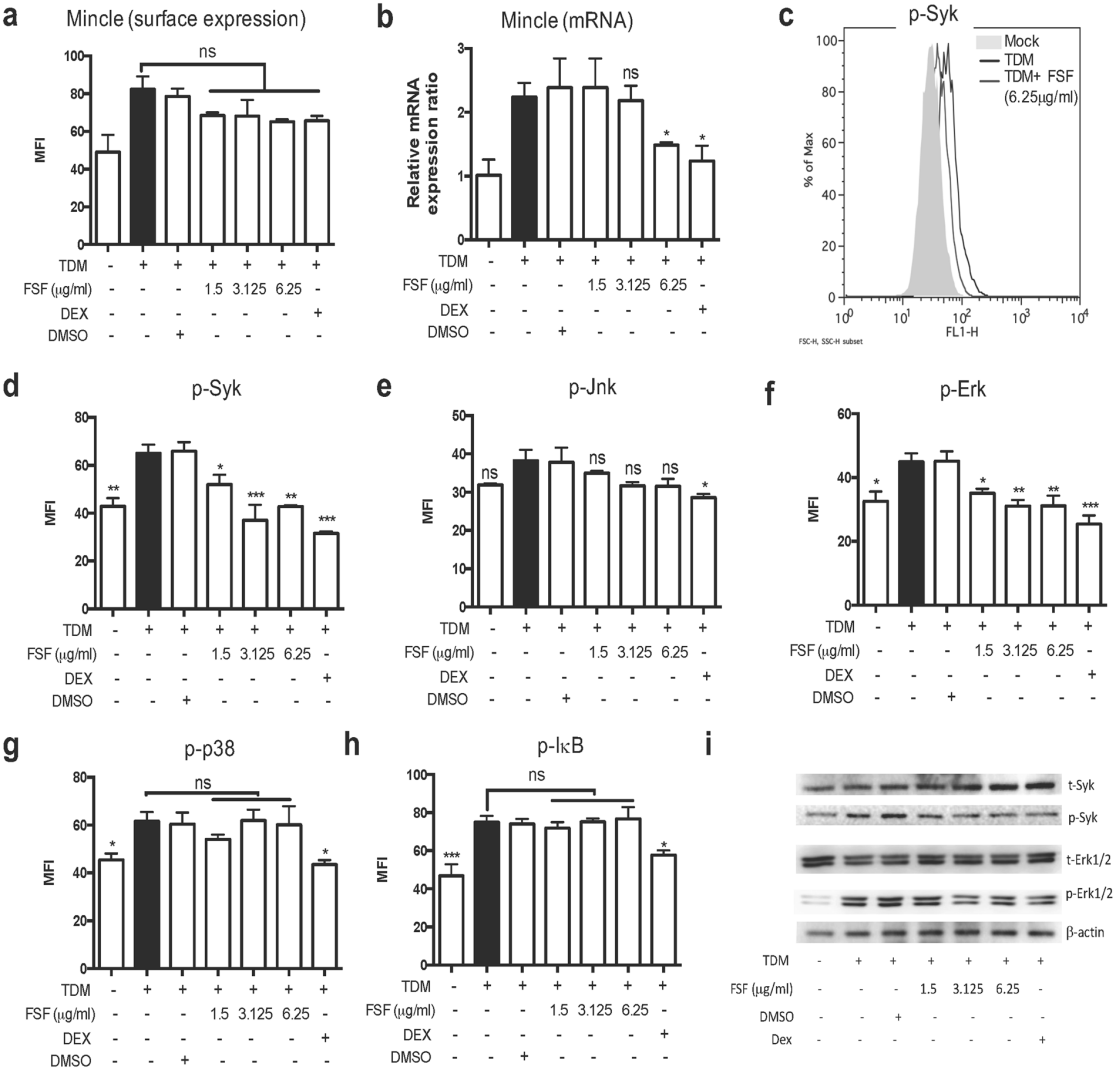
FSF affected Mincle-Syk-Erk signaling pathway in TDM-stimulated MH-S cells. (**a**) Quantification of surface Mincle expression on MH-S cells was presented as MFI. (**b**) The mRNA expression levels of Mincle in MH-S cells were analyzed by real-time quantitative PCR. Values are expressed as the fold of change as compared to unstimulated and untreated control. (**c,d**) Intracellular levels of phosphorylated Syk, (**e**) phosphorylated Jnk, (**f**) phosphorylated Erk, (**g**) phosphorylated p38 MAPK, and (**h**) phosphorylated IκB in cells were measured by intracellular staining and analyzed by flow cytometry. For histogram results of flow cytometry (**c**), grey area represents unstimulated cell staining; blue line represents FSF (6.25 μg/ml) treated cells with TDM stimulation; black line represents TDM stimulation control. For quantification of phosphorylated proteins (**c–h**), results are expressed as MFI and shown as arithmetic mean plus SD of triplicate independent experiments. (**i**) total Syk, phosphorylated Syk, Erk1/2 and phosphorylated Erk1/2 were evaluated by Western blot. Western blot shown was representative of triplicate experiments with essential similar results. The figure is from 5 different gels and the original figure of Western blot is shown in Supplementary Fig. s6. ns, P > 0.05; *P < 0.05; **P < 0.01 and ***P < 0.001 vs. TDM stimulation group.

A previous study has shown that c-type lectin receptor Mincle is a key receptor for TDM (32,33). Mincle is expressed in macrophages and upregulated by TDM stimulation^35^. Syk signaling plays a key role in TDM-induced activation of innate immune cells by engagement of Mincle^36^. Phosphorylation of Syk subsequently activates MAP kinase signaling, leading to the pro-inflammatory responses^35,37^. Other reports also implied the importance of NF-κB signaling in the induction of TDM-mediated inflammation^38^. Therefore, we investigated whether FSF affected these key intracellular signaling mediators on the TDM-induced MH-S activation. Flow cytometric analysis indicated that FSF slightly inhibited TDM-induced surface expression of Mincle (Fig. 6a). In addition, the mRNA expression level of Mincle was significantly suppressed by FSF at 6.25 μg/ml (P < 0.05, Fig. 6b). After FSF treatment (3.125 and 6.25 μg/ml), activation of Syk in TDM stimulated MH-S cells was substantially suppressed (P < 0.001 and P < 0.01, respectively, Fig. 6c and d). MH-S cells showed increased levels of tyrosine phosphorylation of Jnk (Fig. 6e), Erk (Fig. 6f) and p38 MAP kinase (Fig. 6g). However, only Erk phosphorylation was significantly suppressed (Fig. 6f) by treatment with FSF at 1.5, 3.125 and 6.25 μg/ml (P < 0.05, P < 0.01 and P < 0.01, respectively). In addition, no suppression of IκB phosphorylation was observed by FSF administration (Fig. 6h), although IκB phosphorylation was increased by TDM stimulation. Western blot results showed similar suppressive effects of FSF on the phosphorylation of Syk and Erk proteins (Fig. 6i). Together, the results revealed that FSF suppressed the TDM induced Mincle-Syk-Erk signaling pathway activation in alveolar macrophages.

### FSF ameliorated TDM-induced lung granulomatous inflammation

**Figure 7:**
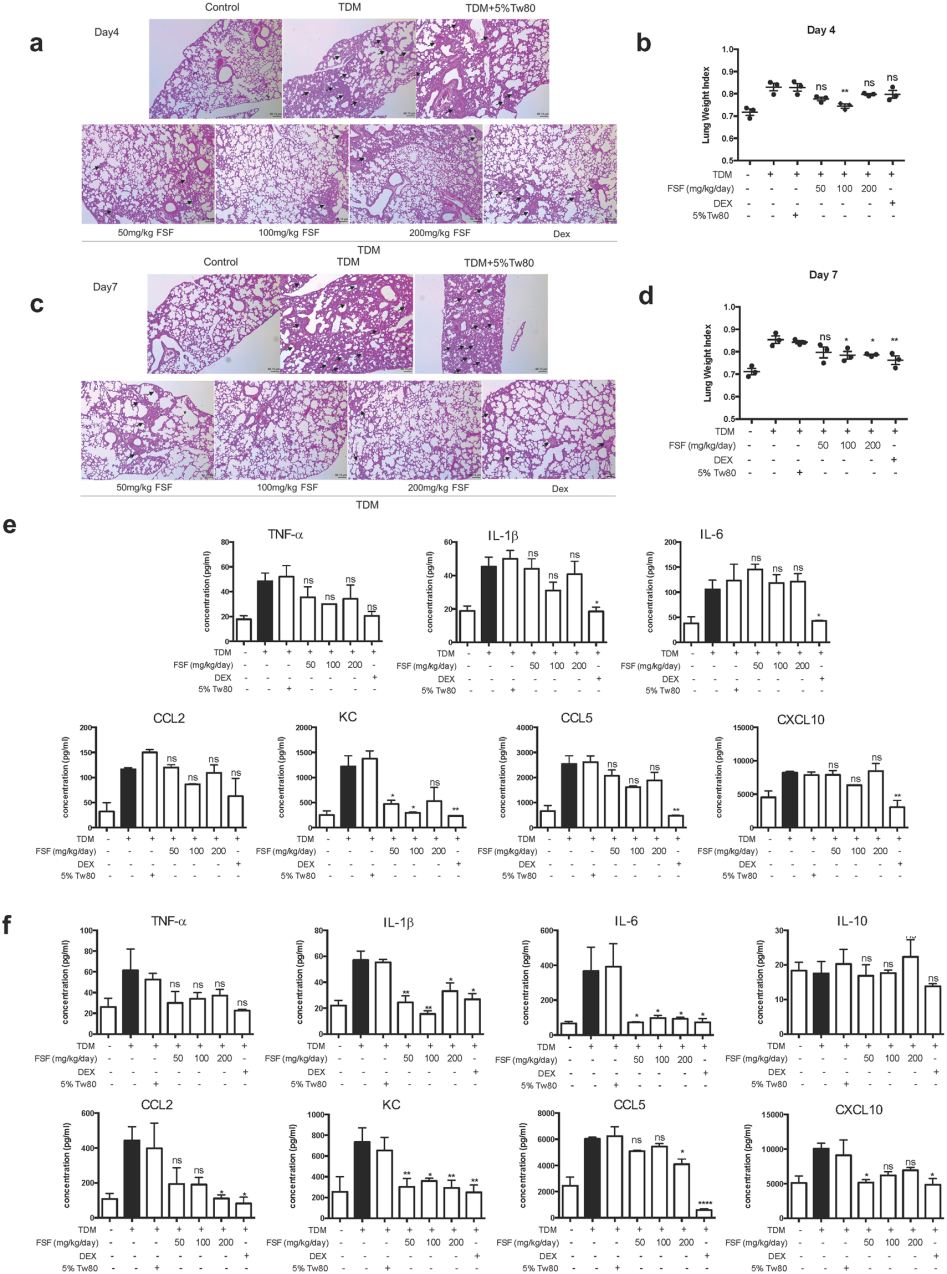
FSF ameliorated TDM induced pulmonary granulomas inflammation in BALB/c mice. Mice were injected intravenously with an oil-in-water emulsion containing TDM. Emulsion without TDM was injected as a vehicle control. Mice were orally administrated with or without FSF for 3 or 6 consecutive days. After 4 or 7 day post TDM challenge, the mice were sacrificed for further analysis. (**a** and **c**) H&E-stained granulomatous response in the lungs of BALB/c mice after 4 (**a**) and 7 (**c**) days of treatment with FSF post challenge by TDM. Arrow heads indicate typical granulomas. Sections are representative of 3 mice per group. (**b** and **d**) Lungs from TDM-challenged mice were removed on day 4 (**b**) and 7 (**d**), and inflammatory intensities were measured by calculating the lung weight index (LWI). n = 3 mice per group. (**e** and **f)** The release of cytokines and chemokines (TNF-α, IL-6, IL-10, KC, CCL5, CXCL10 and CCL2) in murine pulmonary homogenates after 4 (**e**) and 7 (**f**) days of TDM stimulation with or without FSF treatment were quantified by Milliplex assay. Data are representative from 3 independent experiments and expressed as means ± SEMs. Mice (n = 3) were used within each group for different treatments. ns, P > 0.05; *P < 0.05; **P < 0.01 and ***P < 0.001 vs. TDM stimulation group.

It has been reported that intravenous injection of emulsified TDM in mice resulted in the development of granulomas in mice lungs. The number and size of pulmonary granulomas gradually increased in 7 days and slowly resolved afterwards^39^. Therefore TDM-stimulated mice should provide a suitable model for investigating granuloma-related inflammation *in vivo*. In this study, mice were intravenously injected with a TDM oil-in-water emulsion. FSF (50, 100, and 200 mg/kg) were orally administrated to mice for 4 or 7 days. Tween80 (5%) served as solvent control. Mice were sacrificed and samples were analyzed 4 or 7 days after TDM challenge. As illustrated in Fig. 7a, small focal clusters formed by accumulated cells were observed from day 4 after TDM challenge. The clusters became more complex on day 7 (Fig. 7c). Treatment with 5% Tween 80 after TDM challenge showed no significant effects on the symptoms of inflammation and granuloma formation. FSF administration inhibited the inflammation and granuloma induced by TDM in a time- and dose-dependent manner (Fig. 7a and c). Almost no consolidation and granulomas were observed after 7 days of FSF treatment (100 and 200 mg/kg). Orally administration of dexamethasone (0.5 mg/kg) also significantly ameliorated the pulmonary consolidation induced by TDM. TDM-mediated inflammatory lung swelling, as assessed by lung weight index (LWI), was significantly suppressed with FSF treatment for 4 and 7 days at concentrations of 100 and 100/200 mg/kg (both P < 0.05), respectively (Fig. 7b and d).

### FSF suppressed TDM-induced pulmonary pro-inflammatory cytokine/chemokine release in mice

Our *in vitro* study indicated that FSF could suppress the release of pro-inflammatory cytokines and chemokines of TDM-activated mouse alveolar macrophages. We further investigated the expression level of pro-inflammatory cytokines/chemokines, including TNF-α, IL-1β, IL-6, KC, CCL2, CCL5, and CXCL10, in pulmonary tissues from TDM-stimulated mice. As illustrated in Fig. 7e and f, TDM alone significantly induced the secretion of these pro-inflammatory cytokines/chemokines after 4 and 7 days. Most of these cytokines/chemokines were elevated by 2–5 times upon TDM challenge. On day 4, all tested cytokines were suppressed with FSF treatment without significant differences (Fig. 7e). The tested chemokines were also decreased, but only KC showed significant decrease upon FSF treatment (50 and 100 mg/kg, both P < 0.05). However, on day 7, all the tested cytokines except TNF-α, exerted significant decrease (all P < 0.05) under FSF treatment at all tested concentrations (50–200 mg/kg). Suppression of chemokines were also observed in TDM stimulated mouse treated with FSF, and FSF administration (200 mg/kg) showed the most promising suppression effects on KC, CCL2, and CCL5 (P < 0.01, P < 0.05, and P < 0.05, respectively), whereas FSF (50 mg/kg) was the most effective for CXCL10 suppression (P < 0.05). In addition, we also evaluated the expression of regulatory cytokine IL-10 expression in murine lungs after FSF treatment for 6 days. Figure 7f shows that no obvious increase in IL-10 production after TDM challenge was observed, and FSF showed no obvious effect on the expression of IL-10 (all P > 0.05, Fig. 7f). Therefore, these results indicated the suppressive activity of FSF on pro-inflammatory cytokine/chemokine expressions.

### FSF suppressed macrophages and neutrophils infiltration in lung of TDM challenged mice

**Figure 8:**
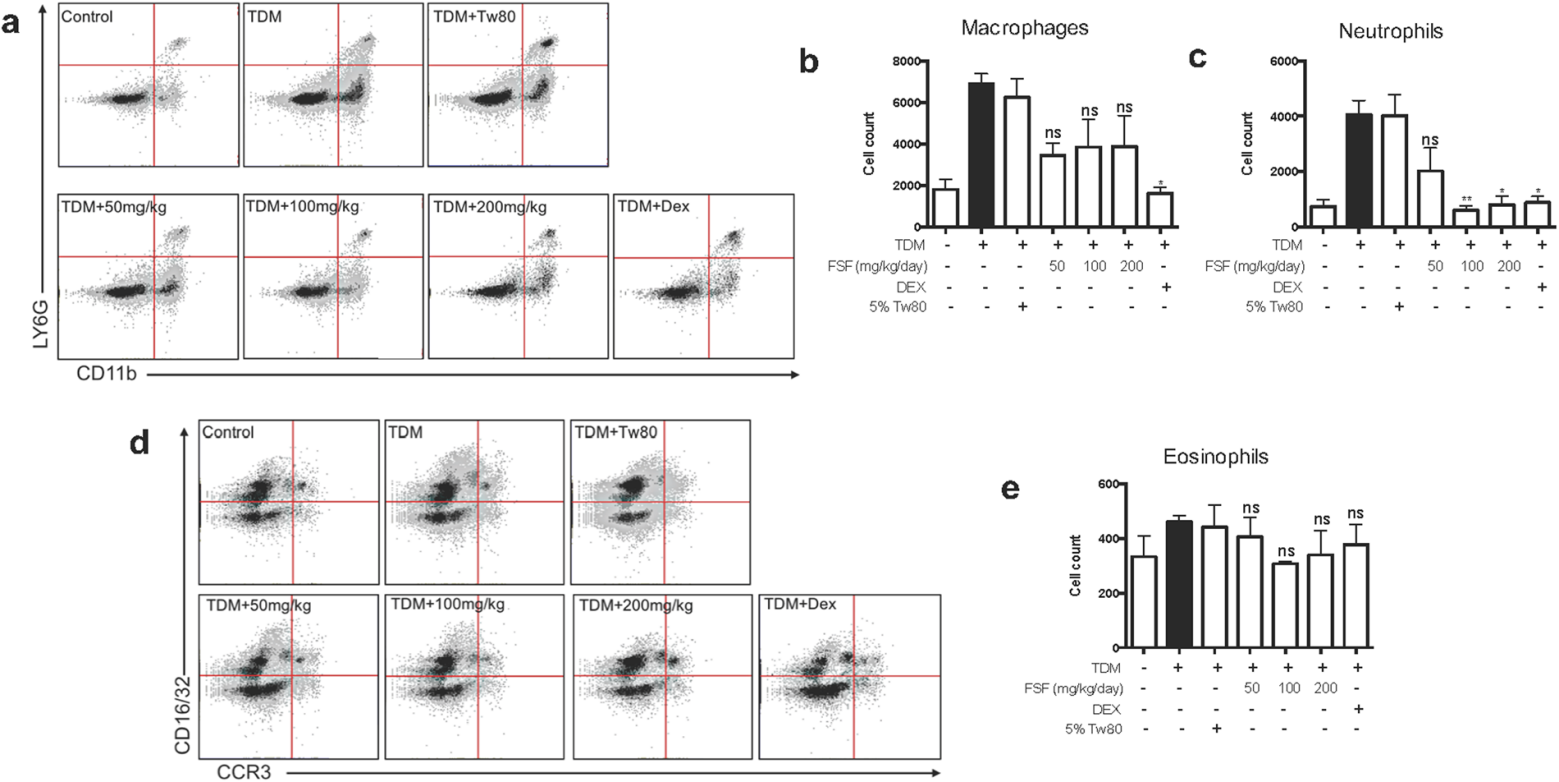
FSF suppressed leukocyte infiltration in TDM-induced lung granulomas inflammation. The leukocyte subsets in lung granulomas after treatment of FSF were evaluated by flow cytometry. Single-cell suspensions were prepared from lung tissues as described in materials and methods. The neutrophils (CD11b+ Ly6G+) (**a** and **c**), macrophages (CD11b + Ly6G−) (**a** and **b**), eosinophils (CCR3 + CD16/32−) (**d** and **e**) were presented in dot plots and bar charts. The bar charts represent quantitative measurement of positively stained cells. Data are representative of three independent experiments and expressed as average ± SEM (n = 3). ns, P > 0.05; *P < 0.05; **P < 0.01 and ***P < 0.001 vs. TDM stimulation group.

For testing the effect of FSF on cell infiltration into pulmonary granulomas, various leukocytes were enumerated in the lungs. Flow cytometric analyses were performed on lung cells sampled on day 7 post TDM change. As illustrated in Fig. 8a–c, the number of macrophages (CD11b+, Ly6G−) and neutrophils (CD11b + , Ly6G + ) were elevated significantly after TDM change for 7 days (all P < 0.05). After TDM stimulation, the numbers of macrophages and neutrophils were increased by 3.5 and 4 times, respectively. After oral administration with FSF at various concentrations, accumulation of macrophages into lungs of TDM-challenged mice were all suppressed without significant differences. In addition, FSF (100 and 200 mg/kg) significantly reduced the infiltrated neutrophils to mouse lungs (both P < 0.01). A previous study has shown that eosinophils may exacerbate the course of mycobacterial infection by the activation of macrophages^40^. We found that the number of eosinophils (CCR3 + , CD16/32-) in the lungs increased slightly after TDM challenge, and no obvious suppression of eosinophils was observed upon FSF treatment at all tested concentrations (Fig. 8d and e). In concordance with a previous report (38), the most dominate leukocyte type in TDM-stimulated mouse lungs was macrophages which in number were about 2 and 15 times of neutrophil and eosinophil counts, respectively (Fig. 8a–c). These results collectively suggest that FSF administration suppressed the leukocyte infiltration, including macrophages and neutrophils, into the TDM challenged mouse lung granulomas.

## Discussion

Effective and optimal host immune response against evasive mycobacterial infection depends on the inflammation and subsequent granuloma formation^41^. However, excessive granuloma-mediated inflammation is also the main cause of detrimental lung injury^2^. Therefore, targeting the pro-inflammatory mediators is considered to be a promising strategy for the treatment of TB. A number of clinical trials using anti-inflammatory treatment combined with standard antibiotic regimens have been proven to exert faster MTB clearance and better clinical outcomes^42^. Herbal medicines have also gained their popularity in the adjunct treatment of TB for their immunomodulatory activities. A previous study has demonstrated the anti-bacterial and anti-inflammatory activities of SF^20^. However, the anti-inflammatory effects and mechanisms of SF, especially its flavonoid fraction (FSF), in TB-mediated granulomas have not been investigated. In the present study, using TDM as a stimulator to mimic the TB mediated granulomatous inflammation, we demonstrated the immune modulatory activity of FSF on TDM-induced inflammation. FSF effectively suppressed TDM mediated murine alveolar macrophages activation via inhibition of the Mincle-Syk-Erk signaling pathway. In addition, FSF treatment reduced inflammation in mouse lungs and infiltration of inflammatory cells, thereby alleviating lung injuries caused by TDM.

Lung alveolar macrophages play a crucial role against tuberculosis infection and the generation of granulomas. The macrophages sense MTB with pattern recognition receptors (PRR), leading to increased cytokine production, inflammatory cell influx and subsequently granuloma formation. In concordance with a previous report^43^, our results showed that TDM significantly induced the production and release of pro-inflammatory cytokines and chemokines from macrophages. FSF significantly suppressed the expression of these cytokines and chemokines without causing any cytotoxicity (Figs 2 and s1a). The lung epithelial cells, which participate in the clearance of infectious agents, are also critical in the protection of respiratory tract by modulating the activity of pulmonary macrophages^44^. In the present study, the release of pro-inflammatory cytokines and chemokines were suppressed by FSF in TDM-stimulated macrophages and MLE-12 cells co-culture with transwell insert (Fig. 3), but not with direct intercellular contact (Fig. s2). However, synthetic steroid dexamethasone could suppress the release of pro-inflammatory mediators under both culturing conditions. Different from dexamethasone, distinct mechanisms might be utilized by FSF. FSF might affect some diffusible mediators released by either lung alveolar macrophages or epithelial cells^27^. However, we cannot exclude the possibility that *in vitro* inflammatory responses were amplified by contact-dependent interactions between alveolar macrophages and epithelial cells^45^, and that FSF could not sufficiently suppress the pro-inflammatory mediators released by contacted co-cultured cells.

Apart from its efficacy in the suppression of cytokines and chemokines in alveolar macrophages, FSF treatment (3.125 and 6.25 μg/ml) substantially inhibited the production of CCL2, chemokine for macrophages, in TDM-stimulated MLE-12 cells (Fig. 4c), thus resulting in the reduction of *in vitro* migration of MH-S cells across transwells (Fig. 4a and b) to MLE-12 conditioned medium. Moreover, FSF significantly suppressed LFA-1 expression, a primary cell adhesion molecule of leukocytes for cellular transmigration into sites of inflammation^46^, on the surface of TDM activated MH-S cells (Fig. 4d and e). These findings collectively suggest that FSF can effectively suppress TDM mediated migration of alveolar macrophages to lung epithelial cells *in vitro*, probably by affecting CCL2 secretion from MLE-12 and/or MH-S cells and LFA-1 expression of MH-S cells.

To further investigate the effect of FSF on TB-mediated inflammation *in vitro*, we employed heat inactivated Bacillus Calmette-Guérin (BCG) for the stimulation of lung alveolar macrophages. Similar results with that of TDM were observed after the FSF treatment. FSF administration suppressed the release of pro-inflammatory cytokine/chemokines expression in BCG activated macrophages (Fig. s3) and transwell co-cultured macrophages/epithelial cells (Fig. s4). FSF exerted no significant effect on the suppression of cytokine/chemokine release of co-cultured macrophages and epithelial cells in a direct contact method (data not shown). However, dexamethasone effectively suppressed cytokine/chemokine release in co-cultured macrophages and epithelial cells regardless of culture methods. Furthermore, FSF also inhibited the expression of LFA-1 (Fig. s5a) and the release of CCL2 (Fig. s5b and c) in BCG activated macrophages, which may contribute to the inhibition of the migration of macrophages. To assess whether FSF affected the anti-microbial activities of macrophages, an intracellular growing *Mycobacterium aurum Tsukamura* (M. aurum) (ATCC) strain were employed. FSF potentiated the killing of bacteria in macrophages (Fig. 5). Taken together, these results indicated the anti-inflammatory and anti-intracellular mycobacterial effects of FSF *in vitro*.

Regarding the intracellular signaling mechanisms, previous reports have characterized the signal transduction pathways of TDM-mediated expression of adhesion molecules and production of pro-inflammatory cytokines and chemokines, implicating an important role of Mincle-Syk, MAPK and NF-κB pathways in the pathogenesis of TDM-mediated granulomatous inflammation^35,36,38,47–49^. Our studies confirmed that Mincle expression was increased upon the stimulation by TDM and both its surface expression and mRNA level were suppressed by FSF administration (Fig. 6a,b). Upon stimulation by TDM, the Mincle downstream molecules Syk and MAPK kinases including p38, Jnk and Erk were all upregulated (Fig. 6c–h). However, unlike dexamethasone, FSF only significantly suppressed the phosphorylation of Syk (Fig. 6c,d and i) and Erk (Fig. 6f and i). This is the first study showing the suppressive effect of FSF on Mincle expression and Syk phosphorylation in TDM-activated macrophages. Our study also demonstrated that TDM could activate NF-κB signaling in murine alveolar macrophages. Different from dexamethasone, FSF showed no apparent suppressive effect on the phosphorylation of IκB (Fig. 6i). Collectively, our results demonstrated that FSF effectively suppressed the Mincle-Syk-Erk signaling pathway in TDM activated alveolar macrophages.

Our *in vivo* experiments using TDM-induced mouse granulomatous model indicated that administration of FSF ameliorated the granulomatous inflammation in murine lungs. FSF inhibited pro-inflammatory cytokine production, pulmonary granuloma formation and inflammation induced by TDM in a time- and dose-dependent manner (Fig. 7). The inhibition was much effective for 7 days treatment with higher dosage (200 mg/kg). In addition, our results showed that the anti-inflammatory cytokine, IL-10, was not significantly affected by TDM stimulation and FSF administration, which is in contrary to the previous studies of the correlation of IL-10 level with the formation of granulomas^50^. In addition to the reduction of lung injury, we observed a decrease in the infiltration of immune cells (Fig. 8). Consistent with a previous report^48^, among the infiltrated cells form granulomas, macrophages were the major part with significantly increased number of neutrophils after TDM stimulation^48^. FSF treatment suppressed the infiltrated macrophages and neutrophils, but not eosinophils (Fig. 8). Furthermore, FSF showed more effective suppression of neutrophils infiltration, thereby indicating a novel role of FSF against granuloma-mediated inflammation. Therefore, our study indicated that FSF ameliorated TDM-induced lung granulomas inflammation in mice without toxic effects (Table s2). The above findings collectively suggest a potential anti-inflammatory role of FSF in TB patients.

In summary, using a TDM-induced inflammation model *in vitro*, we demonstrated that FSF effectively suppressed inflammatory responses of macrophages via inhibiting Mincle-Syk-Erk signaling pathway. Furthermore, we also observed therapeutic efficacy of FSF against pulmonary granuloma-mediated inflammation in mice, thereby implicating a potential therapeutic role of FSF as an adjuvant therapy together with antibiotics for the treatment of TB patients.

## Materials and Methods

### Preparation of FSF

Dried roots of SF were purchased from Beisha Raw Material Medicine Processing Factory (Foshan, China). It was certified for its identity by one of the authors (Ling Cheng) and a voucher specimen (Ref. No. 3540) has been deposited in the herbal bank of Institute of Chinese Medicine, CUHK. FSF extracts were prepared according to previously published method^20^. Briefly, dried roots of SF (300 g) were ground into a fine powder, soaked in 3 L of ethanol, and extracted three times for 3 h at 70 °C. The ethanol extracts were then combined and evaporated to dryness under reduced pressure. The total extract (208.3 g) was suspended in aqueous 2% citric acid solution and partitioned with n-hexane (Sigma-Aldrich Corp., St. Louis, MO, USA) and then ethyl acetate (Sigma-Aldrich) to obtain the alkaloid-free prenylated flavonoid-enriched fraction (FSF, 23.5 g). FSF was used throughout this study. The content of major compounds from ethanol extracts and FSF fraction of SF is listed in Table s1. The extract was evaporated on a rotary evaporator and then stored at −20 °C. For *in vitro* experiments, FSF (500 mg) was dissolved in 5 ml of 50% DMSO (v/v), filtered through a 0.22 μm filter (Falcon, BD Biosciences Corp., San Jose, CA, USA) and diluted with cell culture medium to the indicated concentrations. For *in vivo* experiments, FSF (200 mg) was dissolved in 5 ml of 5% Tween 80 (v/v), filtered through a 0.22 μm filter (Falcon) and diluted with phosphate-buffered saline (PBS) to indicated concentrations.

### High performance liquid chromatography (HPLC) analysis

Chromatographic analysis of Analysis of the ethanol extracts of SF and FSF fraction was performed with Agilent 1100 HPLC system, with data processed using the Agilent OpenLAB CDS Chemstation Edition software (Agilent Technologies, Santa Clara, CA, USA). Separation was performed with a Beckman Coulter Ultrasphere ODS (C18) column (Part No. 235329, 5 μm particle size, 4.6 × 250 mm; Beckman Coulter Corp, Miami, FL, USA) with Alltech Econosphere C18 All-Guard Cartridge (Part No. 96121, 5 μm particle size, 4.6 × 7.5 mm, Alltech Inc., Nicholasville, KY USA) at 30 °C and the injection volume was 10 μl. Gradient elution was performed using solvent A (acetonitrile) and solvent B (0.3% phosphoric acid in 0.3% triethylamine, v/v); the gradient flow was as follows: 0–17 min with 98% B, 17–18 min with 98–50% B, 18–23 min with 50% B, 23–30 min with 50–43% B, 30–31 min with 43–0% B and 31–36 min with 0% B. The flow rate was 1 ml/min, and HPLC chromatograms were obtained using a UV detector at 220 and 290 nm. Standard samples including matrine, sophoridine, oxymatrine, kurarinone, and sophoraflavanone G were dissolved in methanol. Serial dilution was performed to yield final standard concentrations of 6.25, 12.5, 25, 50 and 100 μg/ml.

### Cell culture and *in vitro* treatment

Murine MH-S lung alveolar macrophages and MLE-12 lung type II epithelial cells were obtained from American Type Culture Collection (Manassas, VA, USA) and cultured according to the manufacturer’s instructions. TDM (Sigma-Aldrich) in chloroform (1 mg/ml) was diluted in isopropanol and added into 96-well or 24-well plates at 20 or 50 μg/well, respectively. For stimulation of co-cultured cells with transwells, TDM was separately added to the upper and lower chamber at 20 μg/chamber. The solvent was evaporated in the chemical hood for 5–6 hours at room temperature. For stimulation of cells, cells were seeded onto TDM-coated plates/chambers and incubated for 20 or 48 h. Unless otherwise indicated, FSF, solvent control (0.1% DMSO), or dexamethasone (2 μM, Sigma-Aldrich) were added simultaneously with seeding of cells. For heat inactivated BCG (Shanghai ReBio, China) stimulation, cells were stimulated with heat inactivated BCG (multiplicity of infection (moi) = 10:1) for 20 hours.

### Cell viability assay

MTT (3-(4,5-dimethylthiazol-2-yl)-2,5-diphenyl-2*H*-tetrazolium bromide) was purchased from Sigma-Aldrich. Single cultured MH-S (5 × 10^4^) and MLE-12 (5 × 10^4^) cells were cultured in a 96-well plate with or without FSF treatment for 48 h. The confluent cells were then incubated with MTT (1 mg/ml) for 3 h at 37 °C. DMSO (100 μl, Sigma-Aldrich) was added into each well and incubated at room temperature for complete dissolution of formazan crystals. The plate was read at 570 nm absorbance using a microplate reader (PerkinElmer Corp., Waltham, MA, USA). DMSO at a final concentration (0.01% or less) was included as mock-treated control. The OD readings relative to mock treated samples were plotted.

### Co-culture of MH-S cells with MLE-12 cells

Before co-culture, MH-S cells and MLE-12 cells were cultured separately according to the manufacturer’s instructions. For co-culture using direct intercellular contact method, the medium was changed to RPMI1640 (Gibco Invitrogen Corp, Carlsbad, CA, USA) supplemented with 1% Fetal Bovine Serum (FBS, Gibco, total volume: 500 μl in 24-well cell culture plate), and 3 × 10^5^ MH-S and 1 × 10^5^ MLE-12 cells were co-cultured with or without FSF treatment for 20 h. For co-culture of separated MH-S and MLE-12 cells, 0.4 μm pore size transwell inserts (BD Biosciences) were used. MLE-12 cells were plated at a density of 1 × 10^5^ cells in the lower chamber and MH-S cells were plated at a density of 3 × 10^5^ cells in the upper chamber of transwells. Similarly, the cells were grown in RPMI1640 supplemented with 1% FBS with or without treatment of FSF. Final media volume was 500 μl outside the insert and 200 μl inside the insert. The supernatants inside and outside the insert were collected after 20 h and pooled for cytokine analyses.

### *In vitro* migration assay

MLE-12 cells were stimulated using TDM with or without treatment of FSF for 48 h. Un-stimulated cells were used as control. The culture medium was collected and placed in the lower chamber of transwells. MH-S (3 × 10^5^) cells were loaded into the upper chamber with an 8 μm porous membrane (Corning Inc., PA, USA) in 200 ml RPMI 1640 medium/0.5% FBS and incubated at 37 °C for 24 h. Cells were removed from the upper side of membranes, and nuclei of migratory cells on the lower side of the membrane were stained with crystal violet. MH-S cells that were attached to the filter were counted under light microscopy. Quantitation of migrated cells from at least four randomly selected fields from each slide of three independent experiments was analyzed.

### RNA extraction and quantitative RT-PCR

MH-S cells upon TDM stimulation with or without FSF treatment were frozen in RNAiso plus (Takara Bio Inc., Shiga, Japan). Total RNA was extracted according to the manufacturer’s protocol. cDNA was synthesized using SuperScript II Reverse Transcriptase (Invitrogen). Quantitative real-time RT-PCR analysis of the cDNA was performed using a StepOnePlus Real-Time PCR System (ThermoFisher) with SYBR Select Master Mix (ThermoFisher). The relative mRNA expression of each gene was determined using the ddCt calculation method with GAPDH as the internal housekeeping gene. The primer sequences used were: forward: 5′-GACCAGGTCAAGGTTGTCGTAGAG-3′; reverse: 5′-AGTGAGGCATCAGGTTCAGTCAAG-3′ for Mincle and forward: 5′-TCGCTCCTGGAAGATGGTGATGG -3′; reverse: 5′-GGCAAATTCAACGGCACAGTCAAG-3′ for GAPDH.

### Mice

Inbred male BALB/c mice (8 weeks old) were purchased from The Laboratory Animal Services Centre, The Chinese University of Hong Kong, Hong Kong. The protocol of animal experiments was approved by the Animal Experimentation Ethics Committee of The Chinese University of Hong Kong. All experiments on animals were performed in accordance with the relevant guidelines and regulations outlined in the Animal Experimentation Ethics Committee Guide for the Care and Use of Laboratory Animals, The Chinese University of Hong Kong.

### Induction of pulmonary granulomas through administration of TDM

TDM was prepared as an oil-in-water emulsion to potentiate its immune stimulatory activity. The dry TDM was ground into 9 ml of Freund’s incomplete adjuvant (Invivogen), then homogenized with a 1 ml Tween-80 in 90 ml PBS vehicle. The emulsion was composed of mineral oil (9%), Tween-80 (1%) and PBS (90%). Mice were injected intravenously in the tail vein with 100 ml emulsion containing 40 μg TDM. They were orally administrated with different concentrations of FSF (50, 100 and 200 mg/kg) from the second day of TDM stimulation for 3 or 6 consecutive days. Oral administration of dexamethasone (0.5 mg/kg) and solvent (5% Tw 80) served as controls. Mice were sacrificed at days 4 and 7 post-TDM challenge. Mouse and their lungs were weighed and the calculation of lung weight index was conducted as previously described^51^. The left lungs were fixed in 10% formaldehyde for Hematoxylin and eosin (H&E) staining. The remaining right upper lobes were conserved with ice-cold saline and homogenized. The supernatants were collected for detection of cytokine and chemokines. The right lower lung lobes were processed for flow cytometry analysis.

### Determination of cytokine and chemokine concentrations

Concentrations of murine cytokines including TNF-α, IL-1β, IL-6, IL-10 and chemokines including KC, CXCL10, CCL5 and CCL2 were measured using the mouse cytokine/chemokine Bio-Plex assay kit (Merck Millipore Corp, Billerica, MA, USA) on the Bio-Plex 200 system (Bio-Rad Laboratories, Hercules, CA, USA). For animal experiment, the supernatants of lung homogenous were collected for measurement of cytokine and chemokines.

### Flow cytometry

To measure the surface expression of LFA-1 and Mincle on murine alveolar macrophages, cells were collected by trypsinizing with 0.25% trypsin-EDTA (Gibco), washed with ice-cold PBS, followed by staining with rat anti-mouse LFA-1 antibody (Biolegend Corp., San Diego, CA, USA) or rat IgG1 isotypic antibody (Biolegend) and subsequent Alexa Fluor-488 conjugated goat anti-rat antibody (Biolegend) for LFA-1, and rat anti-mouse Mincle IgG2b (Invivogen) or rat IgG2b isotypic antibody (Biolegend) and subsequent Alexa Fluor-488 conjugated goat anti-rat antibody (Biolegend) for Mincle at room temperature for 30 minutes in the dark. After brief washing with PBS, cells were analyzed with a FACSCalibur flow cytometer (BD Biosciences). For animal experiment, single-cell suspensions were prepared from the right lower lung lobes by cutting into small fragments and digested for 30 min at 37 °C with 2 mg/ml Liberase TL (Roche Life Sciences, Indianapolis, IN, USA) solution. Digested lungs were mechanically disrupted by passage through a sterile strainer (70 μm, Falcon) using the flat portion of a plunger from a 2-ml syringe followed by an additional 70 μm strainer (Falcon). Red blood cells were lysed with 150 mM NH_4_Cl, 10 mM KHCO_3_ and 0.1 mM EDTA. After washing with ice cold PBS, the cells were collected and suspended in PBS, stained with FITC-labeled anti-CD11b and APC-labeled LY6G, PE-labeled anti-CCR3 and APC-labeled anti-CD16/32 (Biolegend). The samples were analyzed on BD FACSVia cell analyzer (BD Biosciences).

### Measurement of phosphorylated signaling molecules by flow cytometry

For the detection of intracellular phosphorylated signaling molecules, TDM stimulated MH-S cells were fixed with a fixation buffer (Biolegend) for 20 minutes in dark at room temperature. After centrifugation, cells were permeabilized with BD Phosflow Perm Buffer III (BD Biosciences) for 30 minutes on ice. After washing 3 times with PBS, cells were stained with PE-conjugated anti-human/mouse phosphor-p38 MAPK (pT180/pY182) antibody (BD Bioscience), PE-conjugated anti-human/mouse phosphor-Erk1/2 (pT202/pY204) antibody (BD Bioscience), eFluor660-conjugated anti-human/mouse phosphor-IκBa (S32/S36) antibody (eBioscience, San Diego, CA, USA) or PE/eFluor660-conjugated isotypic antibodies (eBioscience). Rabbit anti-mouse Syk (p-zap-70(Y319)/Syk(Y352)) antibody (Cell signaling Technology, Danvers, MA) and FITC-conjugated goat anti-rabbit antibody (Biolegend) were used for phosphorylated Syk staining, and anti-mouse p-Jnk antibody (Santa Cruz, Texas, USA) and APC-conjugated goat anti-mouse antibody (R&D systems, Wiesbaden, Germany) were used for phosphorylated Jnk staining. After brief washing with PBS, cells were subjected to flow cytometric analysis.

### H & E staining

Briefly, excised lungs were fixed at 4 °C with 10% formalin for 72 hours, followed by PBS rinsing, dehydrating and paraffin-embedding. Then 4 μm sections were stained with either H&E staining kit (Beyotime Co., Shanghai, China) for the assessment of general morphology and cell infiltration.

### Intracellular killing activity of FSF on M. aurum of lung alveolar macrophages

M. aurum were purchased from ATCC and cultured according to the product instruction. Briefly, M. aurum were cultured at 37 °C in Middlebrook (MB) 7H9 medium (BD Biosciences) enriched with 10% albumin/dextrose/catalase (ADC; BD Biosciences) for liquid growth, and on MB7H10 (BD Biosciences) with 10% oleic acid/ albumin/dextrose/catalase (OADC; BD Biosciences) for solid agar growth. The bacteria in log-phase were maintained as a stock in glycerol (25%) at −80 °C.

For the intracellular bacterial survival assay, macrophages were infected with M. aurum with or without FSF treatment, rifampicin (1 μg/ml) was served as a positive control. After 24 hours, cells were washed and lysed in distilled water at room temperature for 10 min and then scrubbed by a syringe plunger. The lysed cells were spread onto MB7H10 OADC agar plates, and then incubated at 37 °C to determine the colony-forming unit (cfu).

### Western blot analysis of Syk and Erk

Cells were first harvested and lysed with RIPA lysis buffer (Thermofisher Scientific, MA, USA). To avoid protein degradation, protease inhibitor and phosphatase inhibitor (Sigma-Aldrich, St. Louis, MO, USA) were added into lysis buffer. After centrifugation at 15,000 g for 10 min at 4 °C, the supernatant was collected, and the protein concentration was determined with Pierce BCA protein assay (Beyotime, China). Samples containing approximately 50 μg of protein were loaded into each well and separated on 10% SDS-polyacrylamide gels and transferred to polyvinylidene fluoride membranes (Immobilon-PSQ; Millipore, MA, USA). The membrane was blocked with 5% bovine serum albumin (BSA) in Tris-buffer saline/0.1% Tween 20 (TBS-T), and then incubated with the primary antibodies diluted in 5% BSA in TBS-T overnight at 4 °C. The primary antibodies were anti-total syk antibody, anti-phosphorylated syk (p-zap-70(Y319)/syk(Y352)) antibody, anti-Erk1/2 and anti-phospho-Erk1/2 (Cell Signaling Technology, Danvers, MA). The membrane was then rinsed with TBS-T and incubated for 2 h at room temperature with peroxidase (HRP)-conjugated anti-rabbit secondary antibody (Biolegend), diluted in TBS-T with 5% BSA. After washing with TBS-T, the immune complexes were visualized using the enhanced chemiluminescence (ECL) method (GE Healthcare, Piscataway, NJ).

### Statistical analysis

Statistical analyses were performed with Prism software (GraphPad, USA). Differences among groups were assessed by one-way analysis of variance (ANOVA) and were considered significant when the P value was less than 0.05.

## Electronic supplementary material

Supplementary Information
