# Supplementary material for: Sophora flavescens protects against mycobacterial Trehalose Dimycolate-induced lung granuloma by inhibiting inflammation and infiltration of macrophages
Source: Sci Rep. 2018 Mar 2;8:3903. doi: 10.1038/s41598-018-22286-w (PMC5834626; doi:10.1038/s41598-018-22286-w)
Supplement: Supplementary file 1 — Supplementary Information [file 41598_2018_22286_MOESM1_ESM.docx]

***Sophora flavescens* protects against mycobacterial Trehalose Dimycolate-induced lung granuloma by inhibiting inflammation and infiltration of macrophages**

Dehua Liu^1,2^, Ben Chung Lap Chan^1,2^, Ling Cheng^1,2^, Miranda Sin-Man Tsang^1,2^, Jing Zhu^3^, Chun-Wai Wong^1,2^, Delong Jiao^3^, Helen Yau-Tsz Chan^3^, Ping Chung Leung^1,2^, Christopher Wai-Kei Lam^4^, and Chun Kwok Wong^1,2,3,5*^

^1^Institute of Chinese Medicine, The Chinese University of Hong Kong, Hong Kong, China; ^2^State Key Laboratory of Phytochemistry and Plant Resources in West China, The Chinese University of Hong Kong, Hong Kong, China; ^3^Department of Chemical Pathology, The Chinese University of Hong Kong, Hong Kong, China; ^4^State Key Laboratory of Quality Research in Chinese Medicine, Macau Institute for Applied Research in Medicine and Health, Macau University of Science and Technology, Taipa, Macau; ^5^Li Dak Sum Yip Yio Chin R & D Centre for Chinese Medicine, The Chinese University of Hong Kong, Hong Kong, China.

*Correspondence: Professor Chun Kwok Wong, Department of Chemical Pathology, The Chinese University of Hong Kong, Prince of Wales Hospital, Shatin, N.T., Hong Kong. Tel: (852) 3505 2964, Fax: (852) 2636 5090, E-Mail: ck-wong@cuhk.edu.hk

**Supplementary data**

**Table s1**

|  | EtOH Extracts | FSF fraction |
| --- | --- | --- |
| Matrine | 0.5% | UDL |
| Sophoridine | 0.9% | UDL |
| Oxymatrine | 4.8% | UDL |
| Kurarinone | 2.1% | 7.2% |
| sophoraflavanone G | 0.7% | 2.1% |

**Table s1. The content of major compounds from ethanol extracts and alkaloid free flavonoid fraction of SF (FSF).** The content of 5 major compounds in the ethanol extracts and FSF fractions were determined by HPLC analysis at 220 nm for matrine, sophoridine, and oxymatrine, and 290 nm for kurarinone and sophoraflavanone G, respectively. UDL: undetectable level.

**Table s2**

| Treatment | Body weight (g) | | |
| --- | --- | --- | --- |
|  | Day 0 | Day 4 | Day 7 |
| PBS (Tw 80) | 21.7±0.6 | 21.4±0.2 | 22.5±0.7 |
| 50 mg/kg/day | 22.0±0.6 | 21.5±0.3 | 22.4±0.1 |
| 100 mg/kg/day | 21.4±0.1 | 21.8±0.7 | 22.1±0.9 |
| 200 mg/kg/day | 21.6±0.8 | 21.5±0.4 | 22.1±1.0 |
| Dexamethasone | 20.8±0.8 | 20.3±0.2 | 21.3±0.7 |

**Table s2. Body weight of mice administered with FSF.** Each group of Balb/c mice (n=3) were intravenously injected with emulsified TDM (40 μg/mice) and then orally administered with 50, 100 or 200 mg/kg FSF or same volume of saline/dexamethasone daily for 7 days. Body weights were weighed at 0, 4, and 7 days. Data are expressed as mean ± S.D.

**Figure s1**

**Figure s1. Cell viability test for MH-S cells and MLE-12 cells upon FSF treatment.** (a) MH-S cells and (b) MLE-12 cells were seeded on 96-well culture plates and incubated with FSF for 48 h. Cell viability was evaluated by MTT assay. The plate was read at 570 nm absorbance using a microplate reader. The OD readings relative to mock treated samples were plotted.

**Figure s2**

#

IL-6

TNF-α

**Figure s2. The effect of FSF on pro-inflammatory cytokines/chemokines release from co-cultured MH-S/MLE-12 cells (by cell-cell contact) upon stimulation with TDM.** The co-cultured MH-S (3 × 10^5^)/MLE-12 (1 × 10^5^) cells with direct contact were stimulated with TDM and treated with/without FSF or dexamethasone for 20 h. The supernatants were collected for the evaluation of TNF-α, IL-6, KC, CCL2, CXCL10 and CCL5 production of co-cultured cells. DEX, dexamethasone; ns, no significance. Results are shown as arithmetic mean plus SD of triplicate independent experiments. ns, *P < 0.05, **P < 0.01, ***P < 0.001 and ****P < 0.0001 compared with TDM stimulation group.

**Figure s3**

**Figure s3. The effect of FSF on pro-inflammatory cytokines/chemokines release from MH-S cells upon stimulation by heat inactivated BCG.** The supernatants were collected after 20 h incubation and release of CXCL10, IL-6, TNF-α, and CCL5 was determined. DEX, dexamethasone; ns, no significance. Results are shown as arithmetic mean plus SD of triplicate independent experiments. *P < 0.05, **P < 0.01, ***P < 0.001, ****P < 0.0001 and ns vs. heat inactivated BCG stimulation group.

**Figure s4**

**Figure s4. FSF inhibited pro-inflammatory cytokines/chemokines release from co-cultured MH-S/MLE-12 cells (separated by transwells) upon stimulation by heat inactivated BCG.** Secretion of CXCL10, IL-6, TNF-α, and CCL5 by a macrophage/epithelial cell co-culture using transwells (0.4 μm pore size) following by heat inactivated BCG stimulation with or without FSF treatment. The supernatants in the lower and upper chamber were collected, pooled and tested after 20 h incubation. DEX, dexamethasone; ns, no significance. Results are shown as arithmetic mean plus SD of triplicate independent experiments. ns, *P < 0.05, **P < 0.01, ***P < 0.001 and ****P < 0.0001 vs. heat inactivated BCG stimulation group.

**Figure s5**

**Figure s5. FSF suppressed the adhesion molecule (LFA-1) and macrophage chemoattractant protein (MCP-1/CCL2) release *in vitro*.** (**a**) Heat inactivated BCG stimulated MH-S cells were cultured with or without FSF treatment for 20 h. Flow cytometry were then performed after surface staining of LFA-1. Quantitative results of surface LFA-1 expression on MH-S cells are presented as MFI (mean fluorescence intensity). (**b and c**) Secretion of MCP-1/CCL2 in the supernatants from (**c**) MH-S cells and (**d**) co-cultured MLE-12/MH-S cells (separated by transwell) following heat inactivated BCG stimulation with or without FSF treatment for 20 h were determined. Results are shown as arithmetic mean plus SD of triplicate independent experiments. ns, *P < 0.05, **P < 0.01 and ***P < 0.001 vs. heat inactivated BCG stimulation group.

**Figure s6**

β

**Figure s6. The original figure of Western blot in Figure 6i.**
